# Differences in the ventilatory thresholds in treadmill according to training status in 971 males and 301 females: a cross-sectional study

**DOI:** 10.1007/s00421-024-05622-z

**Published:** 2024-09-24

**Authors:** José Antonio Benítez-Muñoz, Pedro J. Benito, Isabel Guisado-Cuadrado, Rocío Cupeiro, Ana Belén Peinado

**Affiliations:** LFE Research Group, Department of Health and Human Performance, Faculty of Physical Activity and Sport Science (INEF), Universidad Politécnica de Madrid, Calle de Martín Fierro, 7, 28040 Madrid, Spain

**Keywords:** VT1, VT2, Oxygen uptake, Heart rate, Running velocity

## Abstract

**Purpose:**

To analyze the influence of training status on the percentage of maximum oxygen consumption, heart rate and velocity (%VO_2max_, %HR_max_ and %V_max_) at which ventilatory threshold 1 and ventilatory threshold 2 occur (VT1 and VT2, respectively), in males and females separately considering age, during a ramp incremental treadmill test.

**Methods:**

791 males (36.8 ± 9.9 years) and 301 females (33.9 ± 11.0 years) performed a ramp incremental exercise test until fatigue where VT1 and VT2 were determined. Participants were classified as low, medium or high training status combining the oxygen consumption at VT1, VT2 and VO_2max_ by clustering analysis.

**Results:**

VO_2max_ is poorly correlated with the %VO_2max_, %HR_max_ and %V_max_ at which VT1 and VT2 occur (*r* < 0.3), in contrast, there is a positive correlation between oxygen consumption at VT1 and VT2 with the %VO_2max_, %HR_max_ and %V_max_ at which VT1 and VT2, respectively, occur in males and females (*r* = 0.203–0.615). Furthermore, we observed the %VO_2max_, %HR_max_ and %V_max_ at which thresholds occur were greater the higher the training status (all *p* < 0.003).

**Conclusion:**

The physiological determinants of the percentage of maximum at which VT1 and VT2 occur are more related to oxygen consumption at VT1 and VT2, respectively, than to VO_2max_. Moreover, due to the higher percentage of maximum at which VT1 and VT2 occur in individuals with a higher training status, the common strategy consisting of establishing exercise intensity as a fixed percentage of maximum might not be effective to match intensity across individuals with different training status.

**Clinical trial registration:**

NCT06246760.

**Supplementary Information:**

The online version contains supplementary material available at 10.1007/s00421-024-05622-z.

## Introduction

Among the most common variables used to prescribe exercise intensity is oxygen uptake (VO_2_). Alternatively, when oxygen uptake measurement is not available, exercise intensity is determined using heart rate (HR) and work rate [power output or velocity (V)]. Traditionally, exercise intensity has been prescribed as a fixed percentage of maximal oxygen uptake (%VO_2max_), maximal heart rate (%HR_max_), and maximum power output (%W_max_) or velocity (%V_max_). These variables are usually expressed relative to their maximum values with the intention of matching intensity between individuals with different training statuses (Mann et al. 2013). It is assumed that exercise performed at the same percentage of maximum produces the same physiological stress across individuals with different training statuses. This strategy is commonly used in recent studies (Coates et al. 2023) and general guidelines (Haskell et al. 2007; Garber et al. 2011).

However, during a graded exercise test, different intensity training zones can be distinguished (moderate, heavy, and severe). These training zones can be identified during an incremental test by determining the first and second thresholds (Meyer et al. 2005; Keir et al. 2015; Weatherwax et al. 2019; Iannetta et al. 2020). The transition from moderate to heavy intensity is indicated by the first threshold, determined through the first lactate or ventilatory threshold (LT1 and VT1, respectively). The transition from heavy to severe intensity is indicated by the second lactate or ventilatory threshold (LT2 and VT2, respectively) (Keir et al. 2022).

Exercise intensity prescription as a fixed percentage of maximum may not be effective if the percentages at which thresholds occur differ between individuals (Meyler et al. 2023). Thus, it is possible that individuals with different threshold percentages could be in different training zones at the same percentage of maximum (Meyler et al. 2023). In this scenario, exercising at the same percentage of maximum would not generate the same physiological stress for everyone due to their differing training zones. An excellent study by Iannetta et al. demonstrated that the percentage of maximum at which thresholds occurred (%VO_2max_, %HR_max_, and %W_max_) varied widely among both males and females, with sex being one of the factors influencing this variability (Iannetta et al. 2020). Despite analyzing males and females separately, they still observed a wide range of percentages at which thresholds occurred (Iannetta et al. 2020). This suggests the potential existence of additional factors contributing to these percentages, highlighting the need to elucidate which factors influence this variation. Contrary to traditional assumptions, they proposed that exercise intensity thresholds do not necessarily occur at higher percentages of VO_2max_ in individuals with higher fitness levels (Iannetta et al. 2020). On the contrary, it has been demonstrated that percentages of VO_2max_ at which thresholds occur are higher in the middle compared to the beginning of the training season, but not significantly different at the end compared to the middle (Zapico et al. 2007). Given these seemingly contradictory findings, it is paramount to determine whether training status affects the percentage of VO_2max_ at which thresholds occur across individuals with varying training statuses, ideally in a study with a large sample size. Moreover, to the best of our knowledge, there is a lack of studies examining differences in the percentage of HR_max_ and V_max_ at which thresholds occur according to training status. If training status impacts the percentage of VO_2max_, HR_max_ and V_max_ at which thresholds occur, intensity could not be set as a percentage of VO_2max_, HR_max_ and V_max_ to standardize intensity across individuals with different training status.

On the other hand, while we acknowledge the existence of other articles with larger sample sizes that provide reference standard values for some of the variables discussed in the present study, these variables are typically stratified by sex and age (Loe et al. 2013; Vainshelboim et al. 2020), but not by training status. Therefore, understanding the reference standard values of these variables according to training status would be valuable for interpreting an individual’s fitness level more accurately. Previous studies have already demonstrated that sex (Iannetta et al. 2020; Vainshelboim et al. 2020), age (Vainshelboim et al. 2020), and ergometer type (Vainshelboim et al. 2020) influence the variables used to determine training zones, suggesting that they should be taken into account when investigating the impact of training status.

Thus, the aim of the present study is to analyze the influence of training status on different variables (VO_2_, HR, and V) expressed relative to their maximum values, used to indicate the first and second ventilatory thresholds (VT1 and VT2, respectively), separately for males and females, while also considering age, during a ramp incremental treadmill test. In addition, we offer reference values for these same variables and additional variables according to training status, leveraging the large sample size.

## Materials and methods

### Participants

This study involved 971 males (75.4 ± 9.6 kg, 175.9 ± 6.7 cm, and 36.8 ± 9.9 years) and 301 females (58.8 ± 7.9 kg, 163.9 ± 5.9 cm, and 33.9 ± 11.0 years) who voluntarily visited our laboratory to undergo an exercise test. Data were collected between 2003 and 2023. Participants were instructed to abstain from vigorous exercise for 2 days prior to the test and to eat at least 2 h before the test. All participants received detailed information about the potential risks and benefits of the study and provided written informed consent to participate. The protocols and procedures were approved by the Ethical Principles for Medical Research Involving Human Subjects of the World Medical Association Declaration of Helsinki (1964) and subsequent amendments. This study was approved by the Human Research Ethics Committee of the Universidad Politécnica de Madrid. Data and information obtained in the project were treated as confidential in accordance with current national legislation governing personal data protection (Organic Law 3/2018). Therefore, access to the database was restricted to researchers involved in the study. Clinical trial registration: NCT06246760.

## Procedures

### Ramp incremental exercise test

The ramp incremental exercise test was performed on a computerized treadmill (H/P/COSMOS 3PW 4.0, H/P/Cosmos Sports & Medical, Nussdorf-Traunstein, Germany). The incremental tests were tailored to each participant’s training experience (sporting background) to accurately determine VT1, VT2, and VO_2max_. Two primary considerations guided the individualization process: first, to avoid excessively long protocols (> ~ 26 min) for well-trained individuals, which could hinder reaching VO_2max_ due to fatigue (Midgley et al. 2008), and second, to prevent overly demanding protocols for poorly trained individuals, which could impede the proper determination of VT1 and VT2 due to protocol duration being too short (Bentley et al. 2007). The test commenced with a three-minute warm-up at a constant intensity between 4 and 6 km/h. Subsequently, the incremental phase involved a ramp test starting from 4 to 8 km/h, with the velocity increasing by 1–1.5 km/h per minute. The incremental phase concluded when the participant could no longer maintain the treadmill velocity. Finally, a 3-min active recovery at the same intensity as the warm-up was followed by a 2-min passive recovery period. Throughout the test, breath-by-breath oxygen consumption (VO_2_) and beat-by-beat heart rate (HR) were analyzed using a Jaeger Oxycon Pro (Erich Jaeger, Viasys Healthcare, Friedberg, Germany) and JAEGER® Vyntus CPX (Jaeger-CareFusion, Hoechberg, Germany), respectively, previously calibrated according to the manufacturer’s specifications.

### Data extracted from incremental tests

VT1 and VT2 were determined as previously published (Rabadán et al. 2011). In brief, VT1 was calculated using: a) the V-slope method, where VT1 represents the breakpoint of the VCO2/VO2 relationship; b) the first exponential increment in ventilation (VE) relative to time; and c) the first increase in the VE/VO2 relationship without accompanying increases in the VE/VCO2 relationship relative to time. VT2 was determined: a) as the second exponential increment in ventilation relative to time; b) as the intensity corresponding to the second increase in the VE/VO2 relationship alongside a concurrent rise in the VE/VCO2 relationship relative to time. VO_2max_ was established as the highest value averaged every 15 s. VO_2max_ was considered achieved if three of the following four criteria were met: voluntary exhaustion of the participant, HR ≥ 95% of the theoretical HR_max_ calculated as 220 minus the age, RER ≥ 1.10, and a plateau in VO_2_ despite an increase in exercise intensity (Poole et al. 2008). All tests were independently evaluated by two researchers, with a third researcher included in cases of disagreement. The following variables were determined: oxygen consumption values at VT1 (VO_2VT1_), VT2 (VO_2VT2_), and maximum (VO_2max_) relative to body weight; percentage of maximal oxygen consumption at VT1 (%VO_2max_ VT1) and VT2 (%VO_2max_ VT2); absolute heart rate at VT1 (HR_VT1_), VT2 (HR_VT2_), and maximum (HR_max_); percentage of maximal heart rate at VT1 (%HR_max_ VT1) and VT2 (%HR_max_ VT2); velocity at VT1 (V_VT1_), VT2 (V_VT2_), and maximum (V_max_); and percentage of maximal velocity at VT1 (%V_max_ VT1) and VT2 (%V_max_ VT2).

### Training status classification

Oxygen consumption at each threshold and maximum was chosen to determine training status for two reasons: (a) its significant impact on health and performance (Poole et al. 2021); and (b) because the exercise protocol appears not to influence VO_2VT1_, VO_2VT2_, and VO_2max_ (Scheuermann and Kowalchuk 1998; Leo et al. 2017; Iannetta et al. 2019), provided the main considerations outlined in the ramp incremental exercise test section are met. A two-step cluster analysis was conducted to determine the optimal number of clusters based on three quantitative variables (VO_2VT1_, VO_2VT2_, and VO_2max_), using the Bayesian Information Criteria (BIC) to identify the clusters with the lowest BIC. In addition, the Silhouette index was used to assess clustering performance, categorized as poor (less than 0.2), fair (0.2–0.5), or good (> 0.5) (Wendler and Gröttrup 2016). The predictor importance of a variable for cluster formation ranged from 0 to 1, derived from a two-step algorithm. Consequently, participants were classified into low, medium, or high training status categories.

### Statistical analysis

All the data are presented as the means ± standard deviation (SD). Outliers were identified as those values that were greater than or less than 3 times the interquartile range. Each outlier was evaluated to decide if it should be removed. The data were tested for normal distribution with the Kolmogorov–Smirnov test and for homogeneity of variances with Levene’s test. All the analysis were performed in males and females independently. Pearson correlation coefficients were calculated to assess the association between VO_2VT1_, VO_2VT2_ and VO_2max_ with the %VO_2max_, %HR_max_ and %V_max_ at which VT1 and VT2 occur. ANCOVA (age as a covariable) was used to study the effect of training status on the different variables determined at VT1 (VO_2VT1_, %VO_2max_ VT1, HR_VT1_, %HR_max_ VT1, V_VT1_ and %V_max_ VT1), at VT2 (VO_2VT2_, %VO_2max_ VT2, HR_VT2_, %HR_max_ VT2, V_VT2_ and %V_max_ VT2) and at maximum (VO_2max_, HR_max_, V_max_). Bonferroni post‐hoc tests were conducted where significant main effects were found in any of the analyzed factors. The effect size of the ANCOVA was calculated by partial eta‐squared (η^2^) and the small, moderate, and large effects corresponded to values equal or greater than 0.001, 0.059, and 0.138, respectively (Cohen 1988). The criteria to interpret the strength of the r coefficients were as follows: trivial (< 0.1), small (0.1–0.3), moderate (0.3–0.5), high (0.5–0.7), very high (0.7–0.9), or practically perfect (> 0.9) (Hopkins et al. 2009). Statistical analyses were carried out with the statistical software Jamovi V1.6 (Jamovi, Sidney, Australia) and SPSS software 29 version (IBM Corp., Armonk, NY, USA). The significance level was set at *p* < 0.05.

## Results

The physical characteristics of the participants and their incremental exercise results are shown in Tables 1, 2 and 3.

**Table 1:** Differences according to training status on the different variables related to oxygen consumption

	Training status	N	Mean	±	SD	Training status effect
Males						
VO_2VT1_ (ml/min/kg)	Low	212	27.4	±	3.9	F(2,961) = 865.13 *p* < 0.001 η^2^p = 0.643
	Medium	436	34.9	±	3.1^a^	
	High	317	42.1	±	3.8^ab^	
VO_2VT2_ (ml/min/kg)	Low	212	38.4	±	3.8	F(2,961) = 1344.9 *p* < 0.001 η^2^p = 0.737
	Medium	436	47.6	±	3.1^a^	
	High	317	57.9	±	4.5^ab^	
VO_2max_ (ml/min/kg)	Low	212	45.0	±	4.4	F(2,961) = 1022.6 *p* < 0.001 η^2^p = 0.680
	Medium	436	54.0	±	3.6^a^	
	High	317	64.0	±	4.7^ab^	
%VO_2max_ VT1 (%)	Low	212	61.4	±	9.2	F(2,961) = 38 *p* < 0.001 η^2^p = 0.073
	Medium	436	64.9	±	6.8^a^	
	High	317	66.0	±	5.9^ab^	
%VO_2max_ VT2 (%)	Low	212	85.7	±	7.7	F(2,961) = 62.7 *p* < 0.001 η^2^p = 0.115
	Medium	436	88.5	±	5.6^a^	
	High	317	90.6	±	4.4^ab^	
Females						
VO_2VT1_ (ml/min/kg)	Low	53	21.3	±	3.4	F(2,289) = 346.44 *p* < 0.001 η^2^p = 0.706
	Medium	161	29.8	±	2.9^a^	
	High	80	37.7	±	3.8^ab^	
VO_2VT2_ (ml/min/kg)	Low	53	30.1	±	4.1	F(2,289) = 427.9 *p* < 0.001 η^2^p = 0.748
	Medium	161	40.5	±	3.2^a^	
	High	80	50.1	±	3.8^ab^	
VO_2max_ (ml/min/kg)	Low	53	34.8	±	3.8	F(2,289) = 380.8 *p* < 0.001 η^2^p = 0.725
	Medium	161	45.0	±	3.1^a^	
	High	80	54.4	±	4.6^ab^	
%VO_2max_ VT1 (%)	Low	53	61.7	±	9.6	F(2,289) = 19.08 *p* < 0.001 η^2^p = 0.117
	Medium	161	66.4	±	6.9^a^	
	High	80	69.4	±	5.9^ab^	
%VO_2max_ VT2 (%)	Low	53	87.4	±	6.1	F(2,288) = 11.91 *p* < 0.001 η^2^p = 0.076
	Medium	161	90.2	±	5.6^a^	
	High	80	92.2	±	4.8^ab^	

VT1 and VT2 ventilatory threshold 1 and 2, respectively; VO_2VT1_, VO_2VT2_ and VO_2max_: relative to body weight values of oxygen consumption at VT1, VT2 and maximum, respectively; %VO_2max_ VT1 and %VO_2max_ VT2: percentage of maximal oxygen consumption at VT1 and VT2, respectively

^a^Significant difference from low training status; ^b^Significant difference from medium training status

**Table 2:** Differences according to training status on the different variables related to heart rate

	Training status	N	Mean	±	SD	Training status effect
Males						
HR_VT1_ (bpm)	Low	212	133	±	13	F(2,961) = 39.1 *p* < 0.001 η^2^p = 0.075
	Medium	436	143	±	12^a^	
	High	317	150	±	12^ab^	
HR_VT2_ (bpm)	Low	212	162	±	13	F(2,961) = 25.9 *p* < 0.001 η^2^p = 0.051
	Medium	436	170	±	10^a^	
	High	317	175	±	10^a^	
HR_max_ (bpm)	Low	212	179	±	11	F(2,960) = 0.558 *p* = 0.573 η^2^p = 0.001
	Medium	435	182	±	10	
	High	317	185	±	10	
%HR_max_ VT1 (%)	Low	212	74.6	±	6.5	F(2,960) = 62.48 *p* < 0.001 η^2^p = 0.115
	Medium	435	78.6	±	5.4^a^	
	High	317	80.8	±	4.8^ab^	
%HR_max_ VT2 (%)	Low	212	90.6	±	5.2	F(2,960) = 72.392 *p* < 0.001 η^2^p = 0.131
	Medium	436	93.5	±	3.4^a^	
	High	317	94.6	±	2.6^ab^	
Females						
HR_VT1_ (bpm)	Low	53	138	±	16	F(2,288) = 5.78 *p* = 0.003 η^2^p = 0.039
	Medium	160	145	±	13	
	High	80	152	±	13^ab^	
HR_VT2_ (bpm)	Low	53	164	±	13	F(2,288) = 2.73 *p* = 0.067 η^2^p = 0.019
	Medium	160	169	±	12	
	High	80	176	±	12	
HR_max_ (bpm)	Low	53	177	±	12	F(2,288) = 1.02 *p* = 0.361 η^2^p = 0.007
	Medium	160	179	±	12	
	High	80	184	±	13	
%HR_max_ VT1 (%)	Low	53	77.9	±	7.1	F(2,288) = 8.98 *p* < 0.001 η^2^p = 0.059
	Medium	160	81.0	±	5.2^a^	
	High	80	82.9	±	4.9^a^	
%HR_max_ VT2 (%)	Low	53	93.1	±	4.1	F(2,288) = 7.47 *p* < 0.001 η^2^p = 0.049
	Medium	160	94.6	±	3.2^a^	
	High	80	95.6	±	2.6^a^	

VT1 and VT2: ventilatory threshold 1 and 2, respectively; HR_VT1_, HR_VT2_ and HR_max_: heart rate at VT1, VT2 and maximum, respectively; %HR_max_ VT1 and %HR_max_ VT2: percentage of maximal heart rate at VT1 and VT2, respectively

^a^Significant difference from low training status; ^b^Significant difference from medium training status

**Table 3:** Differences according to training status on the different variables related to velocity

	Training status	N	Mean	±	SD	Training status effect
Males						
V_VT1_ (km/h)	Low	191	8.7	±	1.4	F(2,798) = 280.33 *p* < 0.001 η^2^p = 0.413
	Medium	371	10.5	±	1.1^a^	
	High	240	11.8	±	1.0^ab^	
V_VT2_ (km/h)	Low	191	12.3	±	1.7	F(2,798) = 324.61 *p* < 0.001 η^2^p = 0.449
	Medium	371	14.5	±	1.1^a^	
	High	240	16.2	±	1.2^ab^	
V_max_ (km/h)	Low	191	15.3	±	1.9	F(2,681) = 117.2 *p* < 0.001 η^2^p = 0.256
	Medium	371	17.0	±	1.4^a^	
	High	240	18.5	±	1.3^ab^	
%V_max_ VT1 (%)	Low	191	57.0	±	8.7	F(2,680) = 43.5 p < 0.001 η^2^p = 0.113
	Medium	371	62.1	±	9.4^a^	
	High	240	64.4	±	5.1^ab^	
%V_max_ VT2 (%)	Low	191	80.7	±	9.3	F(2,680) = 43.5 *p* < 0.001 η^2^p = 0.019
	Medium	371	86.1	±	10.7^a^	
	High	240	89.0	±	5.5^ab^	
Females						
V_VT1_ (km/h)	Low	50	7.2	±	1.4	F(2,256) = 88.7 p < 0.001 η^2^p = 0.409
	Medium	143	9.0	±	0.9^a^	
	High	67	10.4	±	1.1^ab^	
V_VT2_ (km/h)	Low	50	9.7	±	2.0	F(2,255) = 107.8 *p* < 0.001 η^2^p = 0.458
	Medium	143	12.5	±	1.2^a^	
	High	67	14.2	±	1.2^ab^	
V_max_ (km/h)	Low	50	11.4	±	2.3	F(2,229) = 60.4 p < 0.001 η^2^p = 0.345
	Medium	143	14.3	±	1.4^a^	
	High	67	15.4	±	1.3^ab^	
%V_max_ VT1 (%)	Low	50	63.7	±	8.6	F(2,228) = 5.95 *p* = 0.003 η^2^p = 0.050
	Medium	143	63.0	±	6.3	
	High	67	66.5	±	5.3^ab^	
%V_max_ VT2 (%)	Low	50	84.4	±	7.8	F(2,218) = 13.49 *p* < 0.001 η^2^p = 0.110
	Medium	143	86.8	±	6.3^a^	
	High	67	90.9	±	4.9^ab^	

VT1 and VT2: ventilatory threshold 1 and 2, respectively; V_VT1_, V_VT2_ and V_max_: velocity at VT1, VT2 and maximum, respectively; %V_max_ VT1 and %V_max_ VT2: percentage of maximal velocity at VT1 and VT2, respectively

^a^Significant difference from low training status; ^b^Significant difference from medium training status

### Correlations

**Fig. 1:**
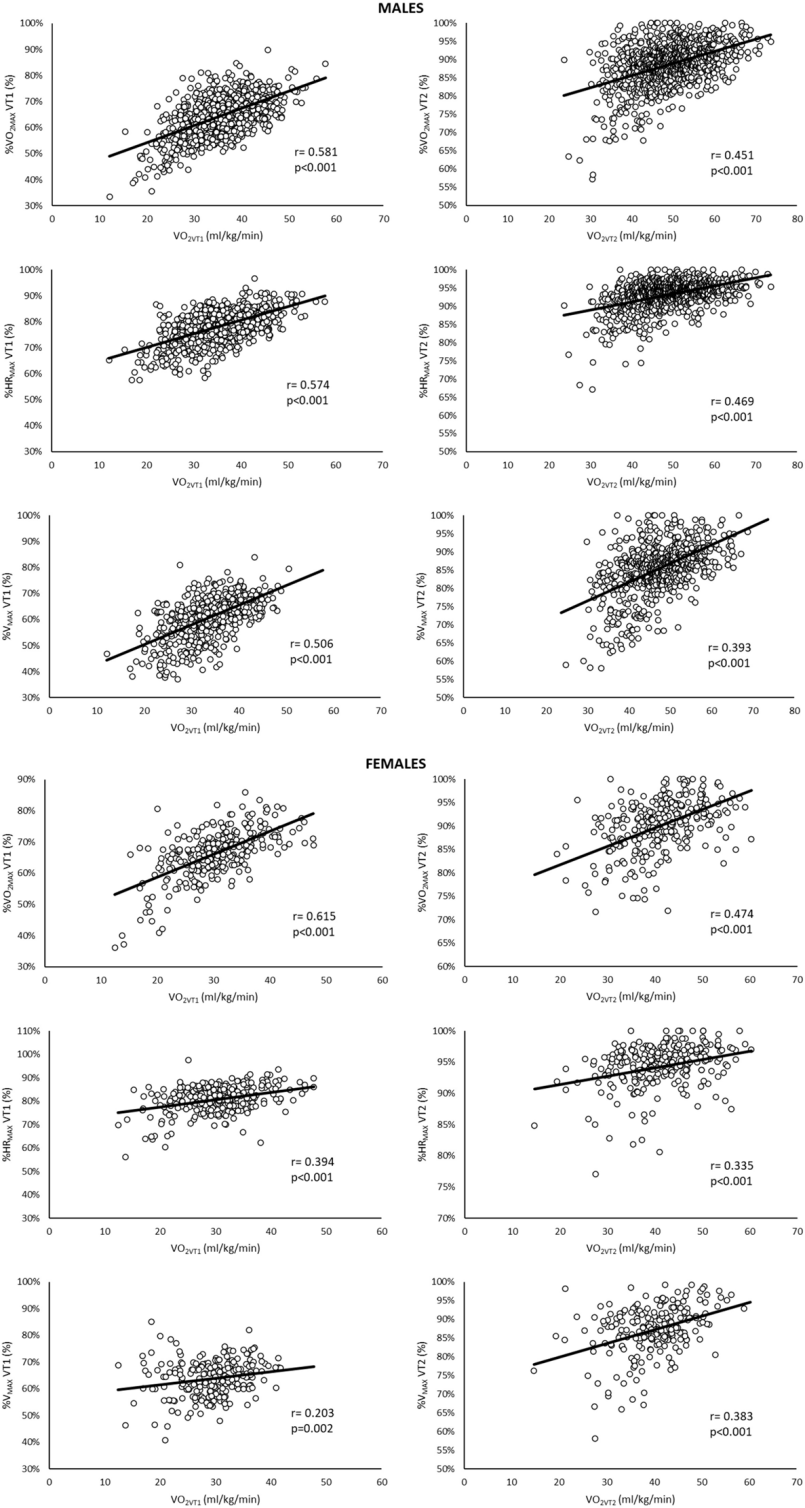
Correlation between VO_2VT1_ and %VO_2max_ VT1, %HR_max_ VT1 and %V_max_ VT1 and between VO_2VT2_ and %VO_2max_ VT2, %HR_max_ VT2 and %V_max_ VT2. VO_2VT1_ and VO_2VT2_: relative to body weight values of oxygen consumption at VT1 and VT2, respectively; %VO_2max_ VT1 and %VO_2max_ VT2: percentage of maximal oxygen consumption at VT1 and VT2, respectively; %HR_max_ VT1 and %HR_max_ VT2: percentage of maximal heart rate at VT1 and VT2, respectively; %V_max_ VT1 and %V_max_ VT2 percentage of maximal velocity at VT1 and VT2, respectively

In males, VO_2max_ was not correlated with the %VO_2max_ VT1 (*r* = −0.029; *p* = 0.364) and %VO_2max_ VT2 (*r* = 0.058; *p* = 0.070) and poorly correlated with %HR_max_ VT1 (*r* = 0.236; *p* < 0.001), %HR_max_ VT2 (*r* = 0.224; *p* < 0.001), %V_max_ VT1 (*r* = 0.108; *p* = 0.005) and %V_max_ VT2 (*r* = 0.112; *p* = 0.003). Similar results were found in females, finding very low correlations between VO_2max_ and %VO_2max_ VT1 (*r* = 0.120; *p* = 0.039), %VO_2max_ VT2 (*r* = 0.128; *p* = 0.028), %HR_max_ VT1 (*r* = 0.186; *p* = 0.001), %HR_max_ VT2 (*r* = 0.192; *p* < 0.001), %V_max_ VT1 (*r* = −0.025; *p* = 0.707) and %V_max_ VT2 (*r* = 0.176; *p* = 0.009). In contrast, a moderate correlation was found between VO_2VT1_ and %VO_2max_ VT1, %HR_max_ VT1 and %V_max_ VT1 and between VO_2VT2_ and %VO_2max_ VT2, %HR_max_ VT2 and %V_max_ VT2 in males and females (Fig. 1).

### Cluster analysis

According to the two-step cluster analysis, the optimal number of clusters was 3 for males and females, categorized as low, medium and high training status. The model’s Silhouette index was 0.5 in males and 0.6 in females, indicating a good quality of the clustering models. VO_2VT2_ had the most importance value (1) followed by VO_2max_ (0.9) and VO_2VT1_ (0.8) in males and in females VO_2VT2_ was also the most important variable (1) followed by VO_2max_ (0.94) and VO_2VT1_ (0.89).

### Training status

%VO_2max_ VT1 and %VO_2max_ VT2 were different according to training status, observing higher values the higher the training status in both sexes. VO_2VT1_, VO2_VT2_ and VO_2max_ were higher the higher the training status of the participant in both sexes (Table 1).

Significant differences were found in %HR_max_ VT1 and %HR_max_ VT2 according to training status in in both sexes. Specifically, in males, both variables increased as training status increased; while in females, %HR_max_ VT1 and %HR_max_ VT2 were higher in high and moderate training status compared to low. HR_VT1_ was higher the higher the training status of the participant in males, but in females, it was only higher in high training status compared to low. HR_VT2_ was higher in high and moderate training status in males, while in females, it was not significantly different across training status. In contrast, HR_max_ was not different across training status in any sex (Table 2).

%V_max_ VT1 and %V_max_ VT2 were significantly different across training status in both sexes. Concretely, in males, both variables were higher the higher the training status. In females, %V_max_ VT1 was higher in high training status compared to moderate and low, while %V_max_ VT2 was higher the higher the training status. V_VT1_, V_VT2_ and V_max_ were higher the higher the training status of the participant in both sexes (Table 3).

## Discussion

The key findings of this study revealed a very weak or no correlation between VO_2max_ and the percentages of maximum at which thresholds occur (%VO_2max_ VT1 and VT2, %HR_max_ VT1 and VT2, %V_max_ VT1 and VT2). However, there was a correlation between VO_2VT1_ and %VO_2max_ VT1, %HR_max_ VT1, and %V_max_ VT1, as well as between VO_2VT2_ and %VO_2max_ VT2, %HR_max_ VT2, and %V_max_ VT2. These results indicate that the physiological determinants influencing the percentage of maximum at which VT1 and VT2 occur are more closely associated with VO_2VT1_ and VO_2VT2_, respectively, rather than with VO_2max_. Moreover, the percentages of VO_2max_, HR_max_, and V_max_ at which thresholds occur were higher in individuals with higher training status. This suggests that the conventional approach of establishing exercise intensity relative to maximum may not effectively standardize intensity across individuals with differing training statuses. These findings contribute to a better understanding of the factors influencing the wide variability of threshold positions and aid in determining exercise intensity based on training zones in individuals with diverse training statuses. Furthermore, the data from this study can serve as standard reference values for these variables in relation to training status, facilitating the interpretation of an individual’s fitness level. The novelty of this work lies in the classification of individuals, which is particularly relevant as detecting the effect of training status on threshold positions necessitates considering oxygen consumption the three main physiological points (VT1, VT2, and VO_2max_). Conversely, if only VO_2max_ is considered, as is traditionally done, no effect of training status on threshold positions is observed. In addition, this novel classification of individuals helps establish reference values for the variables reported in this article according to training status.

In the present study, a very low correlation was found between VO_2max_ and the percentage of maximum at which VT1 and VT2 occur. This could be attributed to the possibility that when specifically training to enhance VO_2max_, improvements in VO_2max_ may outpace those in VO_2VT1_ or VO_2VT2_. In essence, VO_2max_ could be diverging from VO_2VT1_ or VO_2VT2_, thereby reducing %VO_2max_ VT1 and VT2. Conversely, a correlation was observed between VO_2VT1_ and VO_2VT2_ and the position of the respective thresholds relative to VO_2max_, HR_max_, and V_max_. This finding is relevant because relying solely on VO_2max_ fails to capture the impact of training status on other submaximal variables (e.g., %VO_2max_ VT1 and VT2). This indicates that VO_2max_ alone may not adequately distinguish certain critical submaximal variables, such as %VO_2max_ VT1 and VT2. Moreover, the decision to consider VO_2VT1_, VO_2VT2_, and VO_2max_ instead of exclusively utilizing VO_2max_ appears highly pertinent. This is because the oxygen consumption at thresholds has been shown to hold considerable importance for both health and performance, sometimes even surpassing the significance of VO_2max_ (Poole et al. 2021). Therefore, we opted to classify participants by combining VO_2VT1_, VO_2VT2_, and VO_2max_. We provide a practical tool incorporating reference standard values based on specific training statuses at the three physiological points, aiming to aid in participant classification according to training status at these key physiological points (see Figure S1).

In terms of %VO_2max_ at the thresholds, we observed higher %VO_2max_ VT1 and VT2 with increasing training status. This suggests that greater proportional improvements (greater responsiveness) occur in VO_2VT1_ or VO_2VT2_ compared to VO_2max_ as training status improves. We hypothesize that this phenomenon can be attributed to the specificity of training principle, which posits that adaptations primarily occur at the specific intensity at which training is conducted. Given that endurance athletes typically adhere to a pyramidal training intensity distribution (with decreasing training volume from moderate to heavy to severe zones) (Zapico et al. 2007; Casado et al. 2022), it seems reasonable to assume that VO_2VT1_ or VO_2VT2_ experience greater enhancement compared to VO_2max_. An alternative explanation could be the differing impact of genetics on VO_2max_ versus VO_2VT1_ or VO_2VT2_. There is evidence indicating a significant genetic influence on VO_2max_ (Williams et al. 2017), although the impact on VO_2VT1_ or VO_2VT2_ remains unclear. Our findings align with a previous study that observed higher %VO_2max_ VT1 and VT2 in well-trained compared to recreationally trained runners (Hetlelid et al. 2015). Furthermore, the present results are aligned with another study observing that %VO_2max_ VT1 and VT2 differed between professional, U23 and junior cyclist (Alejo et al. 2022). In addition, our results are consistent with another study that noted VT1 and VT2 occurred at higher percentages of VO_2max_ as the training season progressed (Zapico et al. 2007). Contrary, another study found a similar percentage of maximum at which LT occurred between runners of different competitive levels (Støa et al. 2020). In a study by Iannetta et al. (Iannetta et al. 2020), it was specified that the percentage of VO_2max_ at which lactate threshold (%VO_2max_ LT1) and maximal lactate steady state occur (%VO_2max_ MLSS), reflections of VT1 and VT2 when measuring lactate, respectively, did not differ among individuals with different fitness levels. They based this assertion on the lack of correlation between VO_2max_ and the position of the thresholds (%VO_2max_ LT1 and %VO_2max_ MLSS) found in their study (Iannetta et al. 2020). We also observed this lack of correlation between VO_2max_ and %VO_2max_ VT1 and VT2 in the present study. Iannetta et al. suggested that the lack of correlation may be due to the narrow range of VO_2max_ in their study, but this does not seem to be the case because we also found this lack of correlation with a much broader range of VO_2max_ in the present study. In addition, Iannetta et al. (Iannetta et al. 2020) perfectly noted that the lack of correlation could be because the physiological determinants of VO_2max_ are not the same as the physiological determinants of the thresholds. We addressed this issue by evaluating the association between VO_2VT1_ and VO_2VT2_ with %VO_2max_ VT1 and %VO_2max_ VT2, respectively, finding a positive association. These results demonstrate that the physiological determinants of %VO_2max_ VT1 and %VO_2max_ VT2 are more closely related to VO_2VT1_ and VO_2VT2_, respectively, than to VO_2max_. Overall, individuals with a higher specific training status at VT1 and VT2 exhibit a rightward shift in the position of the respective thresholds. Hence, a higher %VO_2max_ VT1 and %VO_2max_ VT2 appear to be adaptations to training. Therefore, we propose them as possible indicators to assess an individual’s progress. However, we do not recommend solely evaluating an individual’s progress or making training decisions based on %VO_2max_ VT1 or VT2, as threshold positions depend on VO_2max_. Thus, a higher %VO_2max_ VT1 or VT2 could result from a lower VO_2max_ with similar VO_2VT1_ or VO_2VT2_, respectively, and this does not necessarily indicate an improvement in training status. The changes in %VO_2max_ VT1 or VT2 with training status observed in our data do not support the recommendations of general guidelines that establish exercise intensity based on percentages of VO_2max_ (Haskell et al. 2007; Garber et al. 2011), since a specific percentage of VO_2max_ could elicit different physiological responses as %VO_2max_ VT1 or VT2 change with training. In other words, at the same percentage of VO_2max_, individuals with different training statuses (different %VO_2max_ VT1 or VT2) could be in different training zones.

Regarding heart rate, we observed higher HR_VT1_ and HR_VT2_ with increasing training status, independently of age. This suggests that HR at the thresholds is sensitive to changes in training status, this interpretation should be taken with caution because this is a cross-sectional study and a longitudinal study would be necessary to confirm this. This finding is the most controversial, as previous studies have reported no significant changes in HR at the thresholds with training over a season (Lucía et al. 2000; Zapico et al. 2007, 2014). One possible explanation is that it may take more than one season to observe changes in HR at the thresholds. If this is true, a single incremental test per season would suffice to determine training zones based on HR data, while additional tests would be necessary to adjust training zones based on HR as training progresses over the years. However, the amount of time needed for training to affect HR at thresholds is currently unknown. Regarding HR_max_, there were no significant differences across training statuses. This result aligns with a previous study that found similar HR_max_ in well-trained compared to recreationally trained runners (Hetlelid et al. 2015). Moreover, it is supported by previous studies that observed similar HR_max_ with training over a season (Lucía et al. 2000; Zapico et al. 2014).

Due to the higher HR_VT1_ and HR_VT2_ and the lack of difference on HR_max_ according to training status, %HR_max_ VT1 and VT2 was significantly higher as the training status increased. This points out that training only can affect HR at thresholds, but not at maximum. These results would indicate that percentage of HR_max_ at which VT1 and VT2 occur increase with training and could be used as an indicator of training status of the participants. This is supported by the significant correlation between VO_2VT1_ and VO_2VT2_ with %HR_max_ VT1 and VT2, respectively. In this case, since training status does not influence HR_max_, it is possible to evaluate the evolution of a participant focusing exclusively on changes in %HR_max_ VT1 and VT2, in contrast to %VO_2max_ VT1 or VT2. Nevertheless, these recommendations should be taken with cautions because the correlation between VO_2VT1_ and VO_2VT2_ with %HR_max_ VT1 and VT2, respectively, was moderate to low in general. As occurred with %VO_2max_, this finding does not support the general guidelines which stablish exercise intensity based on percentages of HR_max_ (Haskell et al. 2007; Garber et al. 2011). Since individuals with different training status (different %HR_max_ VT1 and VT2) could be at different training zones at the same percentage of HR_max_.

V_VT1_, V_VT2_ and V_max_ were significantly higher the higher the training status. These results are aligned with a previous study showing a higher V_VT1_ and V_VT2_ in well-trained compared to recreationally trained runners (Hetlelid et al. 2015). Furthermore, these results agree with previous studies observing an increment in power output at VT1, VT2, and maximum with training during a season (Lucía et al. 2000; Zapico et al. 2007, 2014). Thus, the velocity obtained at the different points seems to be one of the main adaptations to training and deserves special attention when evaluating the evolution of a subject. On the other hand, in case of using velocity to set intensity based on training zones it is necessary to determine velocity at thresholds frequently. This is due to the high impact of training on velocity at the different physiological points.

Finally, we observed higher %V_max_ VT1 and VT2 as the training status of the participants increased. This indicates that greater proportional improvements (greater responsiveness) are observed in V_VT1_ or V_VT2_ compared to V_max_ as training status increases. These results are consistent with those found for %VO_2max_ VT1 and VT2; therefore, they could be explained by the same reasons. %V_max_ VT1 and VT2 could serve as indicators of the training status of the participants due to the higher values observed with higher training statuses. This is supported by the significant correlation between VO_2VT1_ and VO_2VT2_ with %V_max_ VT1 and VT2, respectively, although these recommendations should be interpreted cautiously because the correlation between VO_2VT1_ and VO_2VT2_ with %V_max_ VT1 and VT2, respectively, was generally moderate to low. However, as with %VO_2max_ VT1 and VT2, we do not recommend evaluating the progress of a participant based solely on %V_max_ VT1 or VT2 because a higher value in these variables could be due to a lower V_max_ with similar V_VT1_ or V_VT2_, respectively, and this does not necessarily indicate an improvement in training status. The changes in %V_max_ VT1 or VT2 with training status observed in our data do not support the recommendations of general guidelines that establish exercise intensity based on percentages of maximum work rate, since a specific percentage of V_max_ could elicit different physiological responses as %V_max_ VT1 or VT2 change with training.

### Methodological considerations

Like any study, the present one is not without considerations. One consideration is that participants underwent different incremental ramp exercise protocols depending on their fitness levels. However, various slopes of load increment during the test do not seem to affect VO_2VT1_, VO_2VT2_, or VO_2max_ (Scheuermann and Kowalchuk 1998; Leo et al. 2017; Iannetta et al. 2019), while excessively long protocols (> ~ 26 min) may hinder reaching true VO_2max_ due to fatigue (Midgley et al. 2008). Therefore, it is advisable to avoid an excessively low slope of load increment when assessing a subject’s evolution in VO_2max_. This is the primary reason why participants in the present study did not undergo the same incremental test. Similarly, different slopes of load increment do not appear to affect HR_VT1_, HR_VT2_, or HR_max_ (Scheuermann and Kowalchuk 1998). However, exercise protocols with steeper slopes of load increment yield higher workload values at different physiological points (Jamnick et al. 2018). Regarding reference standard values, other studies report data with larger sample sizes, but they classify individuals based on age and sex rather than training status (Loe et al. 2013; Vainshelboim et al. 2020). Although we acknowledge that our study does not provide data from such a large sample size, to the best of our knowledge, it is currently the only study available in the literature that reports reference values considering training status. Another consideration is that respiratory gas exchange was analyzed using two different analyzers. However, previous studies have already validated the analyzers used in the present study (Rietjens et al. 2001; Perez-Suarez et al. 2018). Finally, like any cross-sectional study, we cannot assert that training itself is the cause of the differences found between training statuses. However, it seems highly impractical to conduct a training program with the sample size of the present study.

## Conclusion

VO_2max_ shows a poor correlation with the percentage of VO_2max_, HR_max_, and V_max_ at which thresholds occur. In contrast, there is a positive correlation between VO_2VT1_ and VO_2VT2_ with the percentage of maximum at which VT1 and VT2 occur, respectively. These results indicate that the physiological determinants of the percentage of maximum at which VT1 and VT2 occur are more closely related to VO_2VT1_ and VO_2VT2_, respectively, than to VO_2max_. This means that VO_2max_ cannot discern some of the differences between different training statuses, such as %VO_2max_ VT1 and VT2. Furthermore, our findings suggest that the %VO_2max_, %HR_max_, and %V_max_ at which thresholds occur were higher with higher training statuses when individuals are classified according to training status combining VO_2VT1_, VO_2VT2_, and VO_2max_. Hence, a higher %VO_2max_, %HR_max_, and %V_max_ appear to be adaptations to training. These results indicate that, despite smaller differences between different training statuses in the variables normalized to the maximum compared to the absolute variables, the common strategy of establishing exercise intensity relative to maximum is not effective in matching intensity across individuals with different training statuses. We offer a practical tool using reference standard values based on specific training status of the three physiological points (VT1, VT2 and maximum) with the intention of helping to classify participant according to training status of the three main physiological points.

## Supplementary Information

Below is the link to the electronic supplementary material.

Supplementary file1 (DOCX 91 KB)

## Data Availability

The data that support the findings of this study are available from the corresponding author, JABM, upon reasonable request.

## Abbreviations

ANCOVA: Analysis of covariance
HR: Heart rate
HR_VT1_: Absolute heart rate at VT1
HR_VT2_: Absolute heart rate at VT2
HR_max_: Maximum heart rate
LT1: Lactate threshold 1
LT2: Lactate threshold 2
MLSS: Maximal lactate steady state
V: Velocity
VCO2: Carbon dioxide production
VE: Ventilation
VO_2_: Oxygen consumption
VO_2VT1_: Oxygen consumption relative to body weight at VT1
VO_2VT2_: Oxygen consumption relative to body weight at VT2
VO_2max_: Maximum oxygen consumption relative to body weight
VT1: Ventilatory threshold 1
VT2: Ventilatory threshold 2
V_VT1_: Velocity at VT1
V_VT2_: Velocity at VT2
V_max_: Velocity at maximum
SD: Standard deviation
η_p_^2^: Partial eta‐squared
%HR_max_: Percentage of maximum heart rate
%HR_max_: VT1 percentage of maximal heart rate at VT1
%HR_max_: VT2 percentage of maximal heart rate at VT2
%VO_2max_: Percentage of maximum oxygen consumption
%VO_2max_: VT1 percentage of maximal oxygen consumption at VT1
%VO_2max_: VT2 percentage of maximal oxygen consumption at VT2
%V_max_: Percentage of maximum velocity
%V_max_: VT1 percentage of maximal velocity at VT1
%V_max_: VT2 percentage of maximal velocity at VT2
%W_max_: Percentage of maximum power output
