# Supplementary material for: Differences in the ventilatory thresholds in treadmill according to training status in 971 males and 301 females: a cross-sectional study
Source: Eur J Appl Physiol. 2024 Sep 24;125(2):499–510. doi: 10.1007/s00421-024-05622-z (PMC11829848; doi:10.1007/s00421-024-05622-z)
Supplement: Supplementary file 1 — Supplementary file1 (DOCX 91 KB) [file 421_2024_5622_MOESM1_ESM.docx]

**Differences in the ventilatory thresholds in treadmill according to training status in 971 males and 301 females: a cross-sectional study**

José Antonio Benítez-Muñoz^1^*, Pedro J. Benito^1^, Isabel Guisado-Cuadrado^1^, Rocío Cupeiro^1^ and Ana Belén Peinado^1^

European Journal of Applied Physiology

^1^LFE Research Group. Department of Health and Human Performance. Faculty of Physical Activity and Sport Science (INEF). Universidad Politécnica de Madrid. Madrid. 28040. Spain. joseantonio.benitez.munoz@upm.es


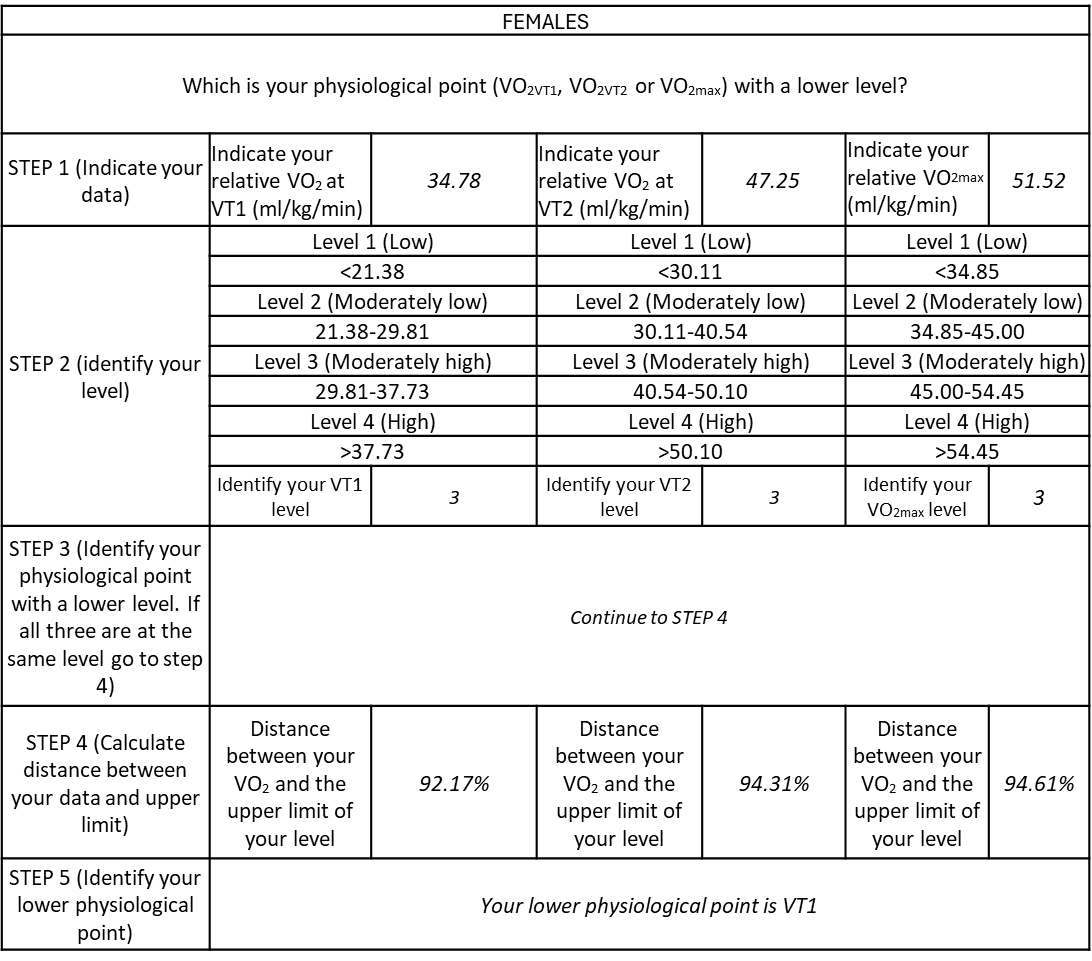

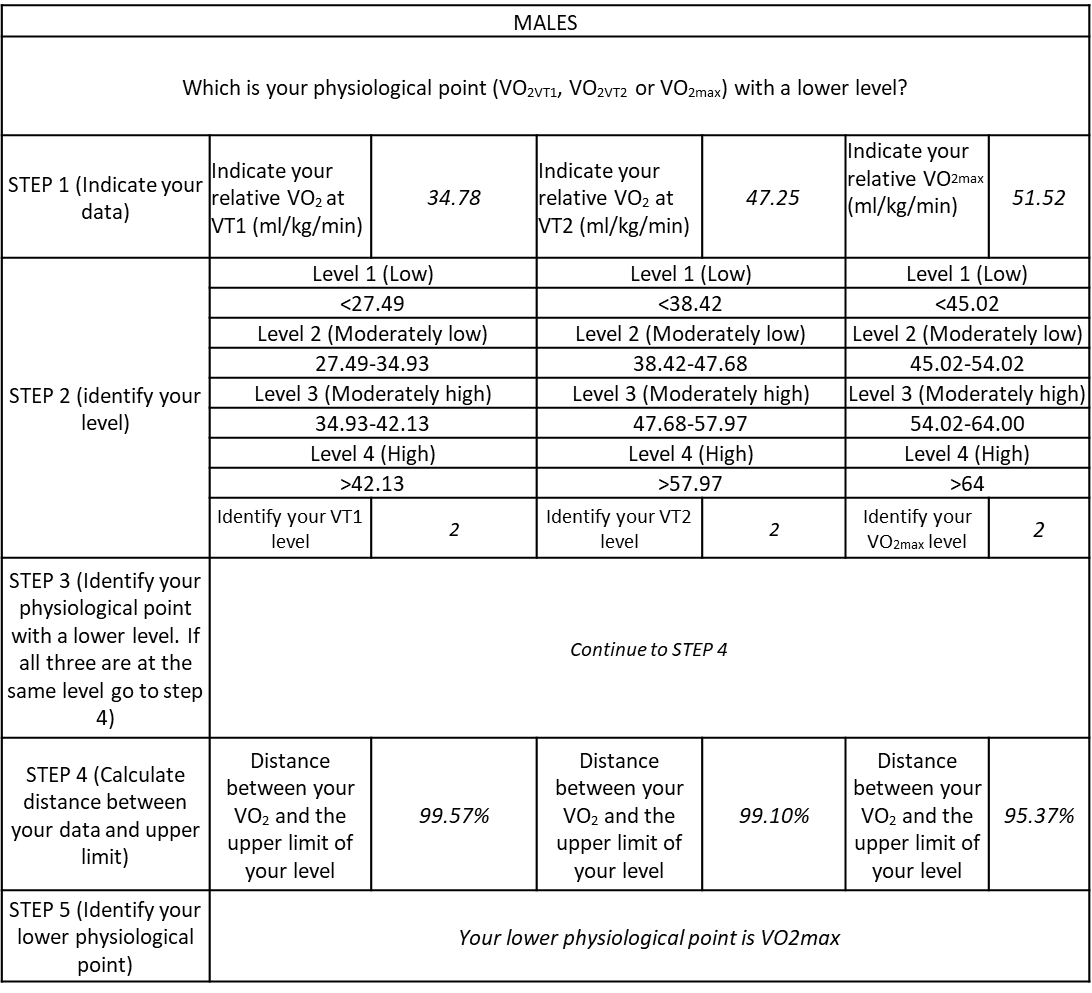


Figure S1. Practical tool to help to classify participant according to training status of the three main physiological points. In italics appears a real example of a participant.
